# PROTOCOL: A comprehensive review of prioritized interventions to improve the health and wellbeing of persons with lived experience of homelessness

**DOI:** 10.1002/cl2.1048

**Published:** 2019-09-12

**Authors:** Kevin Pottie, Christine M. Mathew, Oreen Mendonca, Olivia Magwood, Ammar Saad, Tasnim Abdalla, Vicky Stergiopoulos, Gary Bloch, Vanessa Brcic, Anne Andermann, Tim Aubry, David Ponka, Claire Kendall, Ginetta Salvalaggio, Sebastian Mott, Victoire Kpade, Christine Lalonde, Terry Hannigan, Esther Shoemaker, Alain D. Mayhew, Kednapa Thavorn, Peter Tugwell

**Affiliations:** ^1^ Department of Family Medicine University of Ottawa Ottawa Canada; ^2^ Centre for Global Health Bruyere Research Institute Ottawa Canada; ^3^ C.T. Lamont Primary Health Care Research Centre Bruyere Research Institute Ottawa Canada; ^4^ Department of Epidemiology University of Ottawa Ottawa Canada; ^5^ Physician‐in‐Chief Centre for Addiction and Mental Health Toronto Canada; ^6^ Inner City Health Associates, St. Michael's Hospital University of Toronto Toronto Canada; ^7^ Faculty of Medicine University of British Columbia Vancouver Canada; ^8^ Center for Health and Wellbeing Princeton University Princeton New Jersey; ^9^ School of Psychology University of Ottawa Ottawa Canada; ^10^ Department of Family Medicine University of Alberta Edmonton Canada; ^11^ Faculty of Medicine McGill University Quebec Canada; ^12^ Clinical Epidemiology Program Ottawa Hospital Research Institute Ottawa Canada

## 1 THE PROBLEM, CONDITION OR ISSUE

Worldwide, over 1.8 billion people lack adequate housing and almost 25% of the world's urban population reside in informal accommodation (United Nations Human Rights Council, 2019). “People with a lived experience of homelessness” is a term coined to describe individuals who are, have been, or at risk of becoming homeless. This population lacks stable, permanent, appropriate housing, or may be without immediate prospect, means and ability to acquire it. Such physical living situations can include emergency shelters or provisional accommodations (Canadian Observatory on Homelessness, 2017).

This population continues to grow, giving rise to a major international clinical and public health priority. Homelessness is strongly associated with high levels of morbidity (Hwang, Wilkins, Tjepkema, O'Campo, & Dunn, 2009) and mortality (Nordentoft & Wandall‐Holm, 2003). People with lived experience of homelessness are at an increased risk for acute illnesses, such as traumatic injury (including brain injury), frostbite, peripheral vascular disease, soft tissue infections, and dental decay (Hwang & Bugeja, 2000). Many homeless people also suffer from chronic medical conditions, such as diabetes (Hwang & Bugeja, 2000), cardiovascular disease (Lee et al., 2005), cancer (Krakowsky, Gofine, Brown, Danziger, & Knowles, 2013), and respiratory illnesses (Raoult, Foucault, & Brouqui, 2001). Rates of serious mental illness (SMI; Fazel, Geddes, & Kushel, 2014), cognitive impairment (Stergiopoulos et al., 2015) and drug and alcohol use (Aubry, Klodawsky, & Coulombe, 2012; Grinman et al., 2010; Kennedy, Karamouzian, & Kerr, 2017; Kerr et al., 2009; Torchalla, Strehlau, Li, & Krausz, 2011) are disproportionately high, as are rates of homicide and suicide (Cheung & Hwang, 2004). Moreover, people who are homeless experience a disproportionately high prevalence of infectious diseases, such as Hepatitis C, HIV, and tuberculosis (Beijer, Wolf, & Fazel, 2012; Corneil et al., 2006; Roy, Haley, Leclerc, & Boivin, 2001). These conditions are usually not presented as singular or sporadic incidences as many patients who live in an emergency shelter or on the street experience comorbid conditions and illnesses (Lundy, 1999). However, people with lived experience of homelessness are less likely to access and maintain the care required for their cure and treatment (Milloy et al., 2012; Palepu, Milloy, Kerr, Zhang, & Wood, 2011).

Despite the disproportionally high burden of acute, chronic, and mental illness, people with lived experience of homelessness encounter many barriers to health and social care. The competing need to find food and shelter results in delays in accessing health‐care services (Gelberg, Gallagher, Andersen, & Koegel, 1997). Those who do seek health care often experience discrimination that precludes adequate uptake of preventative health services (Wen, Hudak, & Hwang, 2007), as the structural stigma they experience when accessing health or social services is a major cause of their health inequities (Hatzenbuehler, Phelan, & Link, 2013). For example, people with lived experience of homelessness with coexisting mental health conditions report specific barriers to accessing care, such as being unaware of the location of care, affordability, wait times, and having experienced previous rejection from health or social services (Rosenheck & Lam, 1997). In addition, many health‐care recommendations, such as dietary advice, can prove impossible without access to resources such as proper nutrition and cooking facilities (Hwang, 2001). This lack of appropriate access to community‐based care and reliable social contexts to implement preventive health behaviors results in disproportionately high acute care use by people with lived experience of homelessness (Saab, Nisenbaum, Dhalla, & Hwang, 2016). This population frequently experiences longer hospital stays and a higher risk of unplanned readmission than the general population (Saab et al., 2016), as discharge planning is compromised by inadequate housing to return to and suboptimal structures to support proper follow‐up care (Kushel, 2016).

There is substantial research demonstrating that people with lived experience of homelessness benefit from receiving tailored, patient‐centered care within interprofessional teams with an integrated approach to community and social services (Coltman et al., 2015; Hwang & Burns, 2014; James, Hwang, & Quantz, 2005). A systematic review on health interventions for marginalized and socially excluded populations identified a range of potentially effective interventions that have relevance for marginalized and excluded populations but was not specific to people with lived experience of homelessness (Luchenski et al., 2017). Additionally, numerous studies have looked at the effectiveness of patient‐centered care for people with lived experience of homelessness within community services and social services (Coltman et al., 2015; Hwang & Burns, 2014; James et al., 2005). In order to better understand the needs and resources available for people with lived experience of homelessness, policymakers, practitioners, and allied health professionals need high‐quality systematic reviews and knowledge translation strategies on the effectiveness and cost‐effectiveness of interventions specific to this population. Our review aims to evaluate the evidence on the effectiveness and cost‐effectiveness of interventions that directly or indirectly improve the health of those with lived experience of homelessness.

## 2 THE INTERVENTION

### 2.1 Description of the condition

#### 2.1.1 The intervention and rationale for the interventions

We will evaluate the effectiveness and cost‐effectiveness of interventions for people with lived experience of homelessness aiming to improve their health, health service usage, and social outcomes. Qualitative analysis of service users' intervention acceptability, values, and preferences will be analyzed in a separate review.

We used a Delphi Consensus process (Keeney, Hasson, & McKenna, 2010) to engage 76 people with lived experience of homelessness and 84 health‐care workers and researchers with professional experience in the field of homelessness to rank seven priority topics and needs that are most important for this vulnerable population (Shoemaker et al., 2019). We then scoped literature using Google Scholar and PubMed to determine a list of interventions and terms relating to each of the seven Delphi priority topic categories. Seven priority topic working groups were formed to arrive at a consensus and inform the final selection of the five categories of interventions to be included in this review, based on our literature search. Each working group consisted of medical practitioners, allied health professionals in each area of interest and community scholars (people with lived experience of homelessness or vulnerable housing).

Within this process, issues specific to homeless and vulnerably housed Indigenous Peoples (Inuit, Metis, and First Nations) were identified as a major yet unique priority. Acknowledging this, we are collaborating with an expert team of Indigenous researchers to develop an additional study using participatory methods, which highlights Indigenous‐specific interventions and approaches.

**Figure 1:**
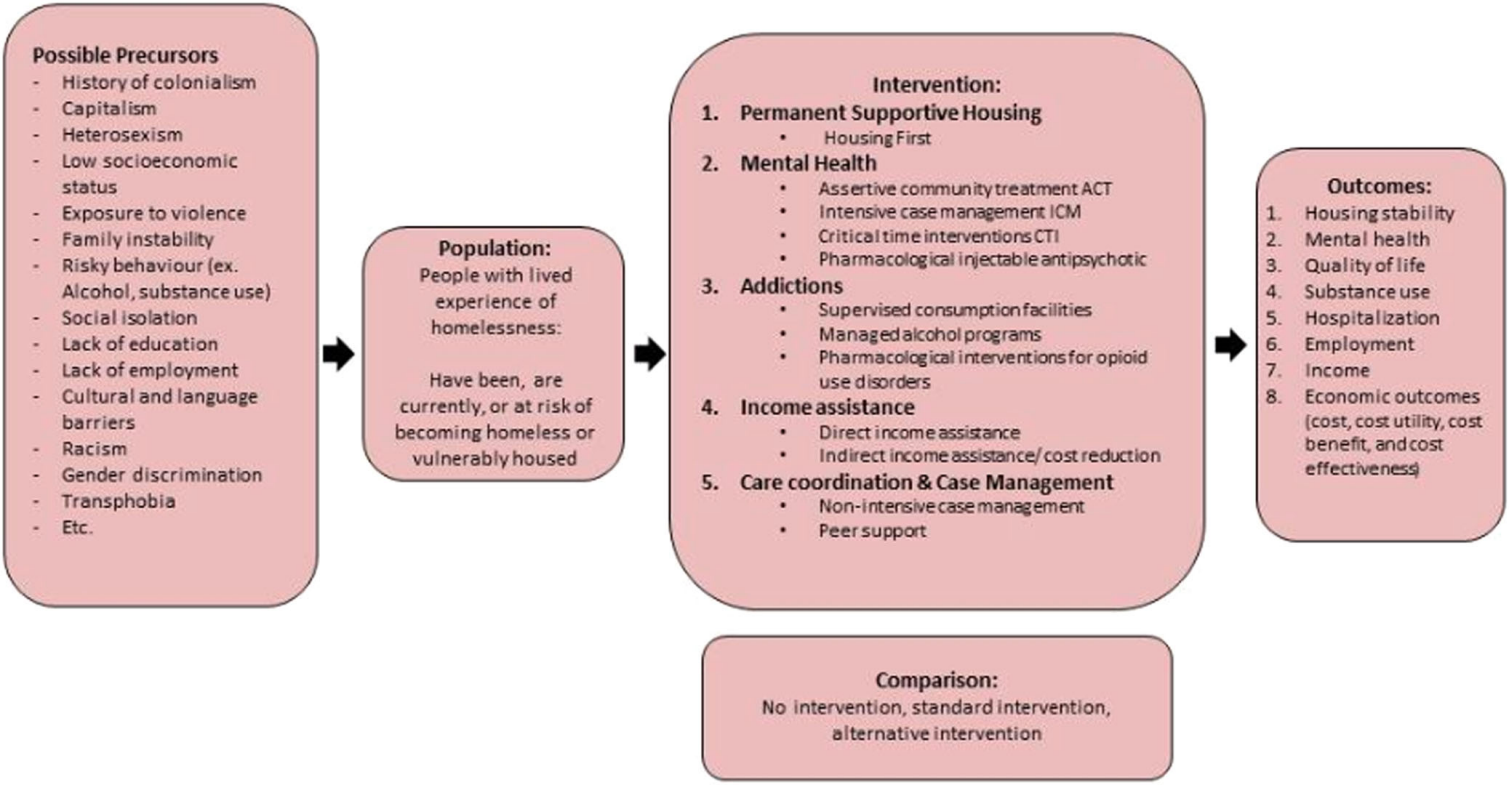
Logic model and project overview

Please refer to Figure 1 for a description of the priority topic categories selected from the Delphi and the related interventions to be included in this review is as follows. Please refer to Appendix A1 for a list of similar interventions that will not be included in this review.

Please refer to Figure 1 Logic Model, PICO project overview.

### 2.2 Description of the intervention

#### 2.2.1 Housing interventions

While examining the effectiveness and cost‐effectiveness of interventions targeting people with lived experience of homelessness, it is important to begin by examining housing interventions available for the targeted population. It is especially dcommon after a period ofifficult for people who are homeless and struggling with mental illness and/or substance use to enjoy housing stability (Schutt et al., 2009). Therefore, many interventions have emerged to help people who are homeless obtain and maintain stable housing (Aubry et al., 2015; Urbanoski et al., 2018). Past housing intervention models prioritized the treatment of mental illness or substance use prior to the provision of housing (Aubry et al., 2016). This course of treatment changed with the emergence of newer permanent supportive housing models developed for homeless populations experiencing mental illness and/or substance use (Aubry et al., 2015; Goering et al., 2011). In these models, such as Housing First (HF), the ability to access housing is not contingent on sobriety/abstinence, or the ability to follow through with treatment plans (Benston, 2015). Rather, in this model the priority is to provide an individual with permanent housing and the choice to access treatment and supports, such as assertive community treatment (ACT) or intensive case management (ICM), offered by a multidisciplinary team (Aubry et al., 2012; Somers et al., 2017). This model acknowledges the critical role housing plays in improving health and social outcomes for this target population. Most studies evaluating the effectiveness of this housing model compared this intervention with no housing or with treatment as per usual.

#### 2.2.2 Mental health interventions

People experiencing homelessness and those who have vulnerably housed experience a high proportion of mental health concerns compared to the general population (Fazel et al., 2014). We will assess three evidence‐supported interventions relevant to SMI, defined as conditions that substantially limit major life activities due to functional impairment (SAMHSA, 2016). These include ACT, ICM, and pharmacological interventions (specifically long‐acting antipsychotics) for psychosis.

##### Assertive community treatment

ACT offers team‐based care by a multidisciplinary group of health‐care workers in the community. This team has 24 hr per day, 7 days per week availability and provides services tailored to the needs and goals of each service user (Coldwell & Bender, 2007; De Vet et al., 2013). There is no time limit on the services provided, but transfer to lower intensity services is common after a period of stability (Homeless Hub n.d.) Ten service users per case manager are typical, and services are offered in a natural setting, such as the workplace, home or social setting (De Vet et al., 2013). The ACT is offered for persons with SMI, often schizophrenia or bipolar disorder, accompanied by a history of multiple psychiatric hospitalizations and functional impairment.

##### Intensive case management

ICM is offered to persons with SMI, but typically fewer hospitalizations or less functional impairment, as well as for people experiencing addictions (Dieterich et al., 2017). ICM helps service users maintain housing and achieve a better quality of life through the support of a case manager that brokers access to an array of services. The case manager accompanies the service user to meetings and can be available for up to 12 hr per day, 7 days a week. Case managers for ICM often have a caseload of 15–20 service users each (De Vet et al., 2013).

##### Critical time interventions

Critical time interventions (CTIs) are a form of time‐limited ICM, defined as a service that supports continuity of care for service users during times of transition; for example, from a shelter to independent housing or following discharge from a hospital. This service strengthens the person's network of support in the community (Silberman School of Social Work, 2017). It is administered by a CTI worker and is a time‐limited service, of usually a period of 6–9 months.

##### Pharmacological interventions for psychosis: injectable antipsychotics

The effectiveness and cost‐effectiveness of injectable antipsychotics among individuals experiencing psychosis will also be examined. This is endorsed as the first line of treatment for psychosis of patients living in precarious situations as they often have poor ability to follow through with oral treatment plans (Llorca et al., 2013). As such it is a viable treatment option for homeless individuals who experience a psychotic illness.

#### 2.2.3 Interventions for substance use

Substance use is disproportionately high among for people with lived experience of homelessness (Palepu et al., 2013). Substance use disorders are typically associated with the recurrent use of alcohol and/or drugs to the point of severe functional impairment (SAMHSA, 2016). Homelessness can be both a cause and result of substance use, and it is important to distinguish occasional substance users from people experiencing a substance use disorder (Vangeest & Johnson, 2002). Once without a home, individuals often experience barriers to accessing treatment for addictions or may experience difficulty following treatment recommendations (Luchenski et al., 2017). We will assess three harm reduction‐focused interventions relating to substance use disorders that apply to people experiencing homelessness and those who are vulnerably housed.

##### Supervised consumption facilities

Supervised consumption facilities (SCFs) are defined as facilities where people who use intravenous drugs can inject preobtained drugs under medical supervision (Drug Policy Alliance, n.d[Link]). Such facilities are frequently used as a safe space for people experiencing homelessness and those who are vulnerably housed as well as substance users. Although these facilities are known to have positive health effects, they have been met with controversy and mixed public opinion (Kolla et al., 2017). We aim to examine the effectiveness and cost‐effectiveness of this intervention.

##### Managed alcohol programs

Alcohol use disorders are more frequent among people with lived experience of homelessness (Fazel, Khosla, Doll, & Geddes, 2008). It is important to ensure that there are helpful interventions in place to assist people living with alcohol use disorders for whom discontinuation of alcohol is not feasible. We will look at the effectiveness and cost‐effectiveness of managed alcohol programs (MAPs) targeting people experiencing homelessness and those who are vulnerably housed. A MAP typically includes shelter, medical assistance, social services and the provision of regulated alcohol to help residents manage alcohol dependence (Shepherds of Good Hope Foundation n.d.). This programme is provided by professional staff and nurses.

##### Pharmacological interventions for opioid use disorders

Opioid use and overdose are on the rise (Doorley et al., 2017) and it is often difficult for users with lived experience of homelessness to access treatment and care. The effectiveness and cost‐effectiveness of opioid therapy medications including, buprenorphine/naloxone, naloxone, naltrexone (British Columbia Centre on Substance Use, 2017) methadone, and injectable diacetylmorphine (heroin; Haasen et al., 2007) have been documented in general population studies. The literature demonstrates the effectiveness of naltrexone (Krupitsky et al., 2011), buprenorphine (with or without naloxone), and methadone (McKeganey, Russell, & Cockayne, 2013), as well as injectable diacetylmorphine (heroin; Haasen et al., 2007) for treating opiate dependence.

#### 2.2.4 Interventions for care coordination and case management

Many people experiencing homelessness and those who are vulnerably housed presently with concomitant mental health, physical health, and social conditions that require coordination of care and access to services. Case management is a myriad of services in which people with multiple morbidities or issues are supported by case managers who assess, plan and facilitate access to health and social services necessary for the person's plan of care and recovery (De Vet et al., 2013). There are many different types of care coordination models which vary according to approach and caseload. The more intensive models of case management, catering to SMI, will be examined under “Mental health interventions”. Here, we will examine two specific areas of less intensive care coordination.

##### Nonintensive case management

The nomenclature for case management is heterogeneous within the literature (Lukersmith, Millington, & Salvador‐Carulla, 2016). As such, we have defined non‐ICM to include two terms: clinical case management (CCM) and standard case management (SCM). Both types of case management allow for the provision of an array of social, health care, and other services with the goal of helping individuals maintain good health and strong social relationships. This is achieved by “including engagement, assessment, planning, linkage with resources, consultation with families, collaboration with psychiatrists, patient psychoeducation, and crisis intervention” (Kanter, 1989). Non‐ICM models are often provided to people experiencing homelessness and those who are vulnerably housed with complex health concerns presenting to primary care practitioners. This is often provided as a time‐limited service.

##### Peer support

Peer support includes the sharing of knowledge, experience, emotional, social or practical help by or with an individual who has experienced a similar background to the service user (Mead, Hilton, & Curtis, 2001). Peer support workers may be termed differently in different settings (i.e., mentors, recovery coaches, life coaches, etc.) but all administrative support to individuals who are newly homeless or on a treatment plan or path to recovery from substance use or homelessness (Barker & Maguire, 2017).

#### 2.2.5 Interventions for income assistance

Financial difficulties or lack of stable income serve as a burden and a barrier to accessing housing, health care, and other needed social supports. Income is essential in order to pay for basic life requirements, such as housing, education, food, clothing, and medication. The provision of income support for people experiencing homelessness and those who are vulnerably housed is a priority as this group can often struggle to attain these necessities. The literature demonstrates that income assistance increases the reported use of health‐care services (Sommers, Blendon, Orav, & Epstein, 2016). There is a need to determine the effectiveness and cost‐effectiveness of income assistance among people experiencing homelessness and those who are vulnerably housed. There are two main interventions for income that we will focus on, direct income assistance and indirect income assistance.

##### Direct income assistance

Direct income assistance consists of benefits and programs offered by individuals or institutions that increase income with the goal of improving socioeconomic status. Direct income assistance can be categorized depending on the regularity of the intervention (i.e., provision of income support on a regular or irregular basis). Some examples include government assistance (i.e., income supplement program (Brownell et al., 2016), charity donation or panhandling (Poremski, Distasio, Hwang, & Latimer, 2015), provision of cheques, tax‐benefits or cash transfers. Cash transfers are a form of financial aid offered on a conditional or unconditional basis (Lagarde, Haines, & Palmer, 2009). Further examples of direct income assistance include support finding and maintaining employment or offering information on income benefits or financial literacy/debt management counseling (Abbott & Hobby, 2000). Direct income assistance is provided by health‐care professionals (social workers or practitioners) and nonprofessionals (community or volunteer organizations) and is provided to people who are: experiencing homelessness, at risk of homelessness, vulnerably housed, have a low income or otherwise socioeconomically disadvantaged.

##### Indirect income assistance (cost reduction support)

The second intervention is cost reduction support, a form of indirect income assistance. This type of intervention includes benefits or programs that improve access to basic living necessities. Examples of specific interventions include the provision of food, daycare, and fuel or rent supplements (Gruber, Chutuape, & Stitzer, 2000; Power, Little, & Collins, 2015; Whittle et al., 2015). This intervention is focused on addressing critical social determinants of health that a person would otherwise be paying for out of their basic income. It is provided by social service, health‐care professionals or nonprofessionals (community volunteers or family members) and provided to people experiencing homelessness, at risk of homelessness, vulnerably housed, have a low income or who are socioeconomically disadvantaged. Indirect income assistance is commonly a time‐limited service during a period of acute need.

### 2.3 How the intervention might work

#### 2.3.1 Housing interventions

Permanent supportive housing, which became known as the HF model, aims to support clients in accessing market housing located among existing rental accommodations, while also providing housing choices for the consumer (Aubry et al., 2015; Somers et al., 2017). This approach provides chronically homeless people who are diagnosed with severe mental illness and/or problematic substance use issues with immediate access to permanent long‐term housing, which can be accompanied by off‐site/mobile support (Stergiopoulos et al., 2015). These flexible support services include ACT or ICM (Aubry et al., 2015). Individuals work with their support team to facilitate access to housing. The prospect of receiving housing is not contingent on abstinence or sobriety, and accommodations are usually available in the form of scattered‐site housing (Aubry et al., 2015). Several positive outcomes have been achieved by scattered housing first (SHF), including residential stability, cost‐saving, improved client satisfaction, quality of life, and reduced severity of psychological symptoms, as well as enhanced social inclusion (community integration) (Somers et al., 2017; Stergiopoulos et al., 2015).

Compared with SHF, congregate format housing, which serves as an alternative option to scattered‐site, is a single supported building (i.e., vacant hotel) where accommodations are reserved for program participants (Somers et al., 2013; Somers et al., 2017). Congregate housing may be located in a residential and commercial area and is equipped with a variety of facilities including a shared central kitchen, recreational areas, and medical services (Somers et al., 2013; Somers et al., 2017). The tenancy is not contingent on sobriety and/or substance abstinence (Somers et al., 2017). In this way, permanent supportive housing focuses on empowerment and autonomy of the individual (Nelson, Aubry, & Lafrance, 2007), which may promote better health outcomes and quality of life in the long‐term.

#### 2.3.2 Mental health interventions

ACT aims to address serious functioning difficulties for those living with a SMI, in areas including “work, social relationships, residential independence, money management, and physical health and wellness” (Stuart, 2009). The literature demonstrates its success in reducing homelessness, improving psychiatric symptom severity, increasing participant interaction with professionals, shortening the length of psychiatric hospital stay, increasing satisfaction with life, and improving community functioning (Coldwell & Bender, 2007; De Vet et al., 2013). These positive outcomes can potentially be attributed to the fact that ACT establishes a genuine and earnest relationship between providers and the service users as the multidisciplinary team makes “a conscious effort to help people avoid crisis situations in the first place or if that proves impossible, to intervene at any time of day or night to keep crises from turning into unnecessary hospitalizations or other negative outcomes”(Stuart, 2009). A separate review of qualitative studies will further examine the relationship between service users and providers and their impact on program effectiveness (Magwood et al., 2018).

Literature shows that ICM can improve economic security (De Vet et al., 2013), reduce hospitalization and increase retention in care for people with SMI in comparison to standard care (Dieterich et al., 2017). This can be attributed to linking the service user to appropriate supports within the community and increasing access to services, home visits, and education (Coles & Coles, 2017).

Also, evidence on CTIs among homeless populations demonstrates an improvement in housing stability, improved psychopathology decreased psychiatric hospitalization, and cost‐effectiveness (De Vet et al., 2013). Indeed, CTI improves the continuity of care by strengthening a service user's ties with services, family, and friends, contributing positively to a decreased number of nights spent homeless during periods of transition (Jones et al., 2003).

Lastly, long‐acting Injectable antipsychotics are effective for managing numerous serious mental disorders, such as schizophrenia, delusional disorder, and bipolar disorder (Llorca et al., 2013). This treatment is mentioned as the first line of treatment for subjects in precarious situations, as they often have poor ability to follow through with oral treatment plans, making it a viable and effective option for people experiencing homelessness and those who are vulnerably housed.

#### 2.3.3 Addictions interventions

Interest in SCFs is increasing as evidence demonstrates that implementation of such facilities is cost‐effective (Kennedy et al., 2017), linked to an increase in detoxification service use, and has increased rates of long‐term addiction treatment initiation (Kennedy et al., 2017; Wood, Tyndall, Zhang, Montaner, & Kerr, 2007). These benefits can be attributed to the fact that SCFs provide low barrier accessible and nonjudgmental services, connect people who use drugs with health and social services and provide a safe user environment (Kennedy et al., 2017). Evidence on the effectiveness of MAPs among people experiencing homelessness and those who are vulnerably housed demonstrates their ability to help stabilize alcohol intake and significantly reduce encounters with police and emergency department visits (Podymow, Turnbull, Coyle, Yetisir, & Wells, 2006). MAPs prioritize the treatment of people experiencing homelessness and vulnerable housing that have severe alcohol use disorders and who may have been denied access to other abstinence‐based or harm reduction programs. Uniquely, it provides service users with shelter, regulated alcohol dispensing, medical care, and support from health‐care professionals; these supports allow for a reduction in harms among this population (Podymow et al., 2006). Lastly, the use of pharmacological interventions will be examined in relation to opioid use disorders. The literature demonstrates that opioid agonist treatments, such as methadone, buprenorphine/naloxone, and oral morphine are more effective than withdrawal management alone and reduce the risk of morbidity and mortality (British Columbia Centre on Substance Use, 2017) and provide a stable alternative to previously unpredictable drug supply. Opioid antagonist treatments such as naltrexone have been documented in overdose treatment and some evidence demonstrates reduced reincarceration rates (British Columbia Centre on Substance Use, 2017) among general populations.

#### 2.3.4 Care coordination and case management interventions

Though case management interventions are heterogeneous in nature and complexity (Lukersmith et al., 2016), studies describing SCM and CCM found that housing retention and substance use outcomes improved (De Vet et al., 2013), and that these interventions are associated with service users feeling more supported and guided by the provision of service coordination (Conrad et al., 1998). Additionally, literature reported a reduced frequency of emergency department use and reduced costs due to an increase in access to other services such as primary care (Shumway, Boccellari, O'Brien, & Okin, 2008).

Previous studies on Peer support in people for people experiencing homelessness and those who are vulnerably housed found that role modeling, shared experiences, and social support significantly impact the perceived quality of life as well as improvements in social support and drug and alcohol usage (Barker & Maguire, 2017).

#### 2.3.5 Income support interventions

The health benefits of income assistance programs lie in their ability to improve the socioeconomic status, reduce severe financial stress, and reduce barriers to meeting essential costs of living such as housing, food, clothing, and medication (Richards, Cohen, & Klein, 2008). In examining existing literature, a powerful body of evidence demonstrates that higher income and social status lead to better health (Government of Canada, 2019; Lynch et al., 2004). For low‐income individuals, Income assistance reduces financial strain, improves psychological status, and quality of life, and may play a role in reducing future morbidity and mortality (Abbott & Hobby, 2000). Increased income improves access to preventative and curative health services and may be an effective strategy to improve health status (Lagarde et al., 2009). Income assistance programs may also improve dietary diversity and nutrition health services utilization (Lagarde et al., 2009). Furthermore, recipients of information relating to income benefits may have improved physical and psychosocial health (Adams, White, Moffatt, Howel, & Mackintosh, 2006). By improving the socioeconomic status, interventions for income security may lead to improved psychological, physical, and social health.

### 2.4 Why it is important to do this review

There is substantial research demonstrating that people experiencing homelessness and those who are vulnerably housed benefit from receiving tailored care from interprofessional teams with an integrated approach to community and social services (Coltman et al., 2015; Hwang & Burns, 2014; James et al., 2005). We believe our review will be unique as it will report on a wide range of health and social service outcomes for our interventions of interest, such as housing stability, mental health, quality of life, substance use, hospitalization, employment, and income‐related outcomes. We believe that in order to facilitate the provision of appropriate care and foster knowledge translation and policies within this area, it is important to assess interventions on a range of outcomes in order to better understand their effectiveness. Despite ample literature in this field, not many reviews exist which comprehensively analyze interventions for numerous health and social outcomes. Another added value of our work is that it will report on findings from distinct interventions relevant for this population; an approach that is challenging in concept but rewarding in results.

Below we summarize systematic reviews that are relevant to our study population and explain the importance of our review.

#### 2.4.1 Health interventions for people who are homeless (Hwang & Burns, 2014)

Hwang and Burns's review outlines interventions to improve the health of homeless people with practical suggestions for health‐care providers working with this population, from the two literature searches yielding 21 systematic reviews of interventions and 58 articles. Housing provision with mental health support was reported to be superior compared to mental health care alone. Based on the results, the authors concluded that interventions needed to be tailored to the circumstances of homeless populations for better outcomes. There is a sparsity of studies on the subjective perspectives of those experiencing homelessness and their view of mental health care service provision and overall documented experiences were negative. According to the authors, the health‐care providers should understand that an effective strategy to address homelessness will need to include both interventions to improve the health of homeless individuals and larger‐scale policy changes and interventions directed at these structural factors. The review did not include studies targeting income support which we believe to be an important intervention for this population. The search strategy ended on December 2013, allowing us to retrieve any studies published after this date.

#### 2.4.2 Interventions to improve the health of the homeless (Hwang, Tolomiczenko, Kouyoumdjian, & Garner, 2005)

Hwang, Tolomiczenko, Kouyoumdjian, and Garner performed a systematic review of interventions for populations experiencing homelessness or vulnerable housing conditions. These interventions were provided by primary care services or to which patients experiencing homelessness and those who are vulnerably housed could be referred. In this review, 73 studies (RCTs and longitudinal) were included. The authors categorized findings based on subgroups experiencing homelessness: individuals with mental illness, with substance use, with concurrent mental illness and substance abuse, with tuberculosis, youth, families and children, women, those at emergency departments or admitted to hospitals. The authors concluded that in comparison to usual care, there are improvements in health outcomes through the provision of coordinated treatment and support for adults experiencing homelessness with mental illness and/or substance abuse. This study was conducted over 10 years ago. The study does not look at income interventions nor does it report on social outcomes such as employment, income, and access to services. The study reports that doing a cost analysis was beyond its scope, which we hope to address in our review.

#### 2.4.3 Effectiveness of interventions to reduce homelessness (Munthe‐Kaas 2016)

The authors of this systematic review looked at the effectiveness of housing and case management programs for people experiencing homelessness and who are at risk of becoming homeless. The main outcomes assessed were housing stability and reduction in homelessness. All relevant databases and sources of grey literature were systematically searched. This systematic review identified critically appraised and synthesized evidence from 43 randomized controlled trials. No restrictions were made in terms of language, location or time. Studies were searched until January 2016. The certainty of findings was assessed using the Grading of Recommendations, Assessment, Development, and Evaluation (GRADE) approach, and included studies varied from very low to moderate certainty. The review found that CTI, abstinence‐contingent housing, nonabstinence‐contingent housing with high ICM, housing vouchers, and residential treatment were effective compared to usual care. This review reports on exclusively on housing outcomes, whereas our review will also assess health and social outcomes as well, such as quality of life, substance use, employment, and income. Additionally, we will review other interventions besides case management and HF, and include a broader range of study designs besides solely randomized controlled trials.

#### 2.4.4 A review of the literature on the effectiveness of housing and support, ACT, and ICM interventions for persons with mental illness who have been homeless (Nelson et al., 2007)

Nelson et al. (2007) performed a literature review examining housing and support interventions for people with mental illness who are homeless. The review included 16 controlled outcome evaluations. The included studies met all the following criteria: published in refereed journals, experimental or quasi‐experimental in design, focused on people with mental illness who had a history of being homeless, measured outcomes quantitatively, included supportive housing, ACT or ICM as the intervention. Studies were searched until December 2004. Reported outcomes included: housing stability, quality of life, and well‐being, psychosocial and mental health, clinical and service use. The authors found that the most effective interventions were those that combined housing and support, and the weakest was providing ICM alone. The scope of this study is limited to interventions for homeless populations that target housing and supportive interventions and does not report on various other health and social outcomes.

#### 2.4.5 Effectiveness of interventions to improve the health and housing status of homeless people: a rapid systematic review (Fitzpatrick‐Lewis et al., 2011)

Fitzpatrick‐Lewis et al. (2011) conducted a rapid systematic review to examine interventions that improve the health and housing status of homeless people. Included studies (n = 84) had to examine the effectiveness of an intervention to improve the health and health care utilization of people who were homeless, marginally housed, or at risk of homelessness and had to be published between 2004–2009. Interventions were categorized by subgroup into: as those for people with mental illness, substance abuse, concurrent mental illness and substance abuse, people with HIV, youth, women, or families, and children. This rapid review synthesizes existing evidence on interventions that improve health, housing status, and access to health care for homeless populations. Our review will assess the effectiveness of interventions on a wider scope of outcomes including quality of life, employment, and income.

#### 2.4.6 How can health services effectively meet the health needs of homeless people? (Wright & Tompkins, 2006)

Wright and Tomkins (2006) review and synthesize evidence on the effectiveness of health care interventions for homeless populations. The authors searched all main databases (Medline, EMBASE, CINAHL, PsychINFO, and Cochrane), as well as grey literature and expert opinion. The search was not limited to language or geography, and publication dates searched ranged from 1980–2003. The authors did not conduct a meta‐analysis of data. As such, the articles were synthesized by recurring themes: morbidity, mortality, primary care provision, primary prevention interventions, management of drug dependence, medically supervised injecting centers, sexual health promotion, management of alcohol dependence, and management of mental illnesses. Each theme described relevant interventions and linked to the main diseases common among the population. Included studies were not critically appraised for quality or certainty. Our review will focus on different interventions such as permanent supportive housing and case management programs to assess their effectiveness on a wider scope of outcomes, including but not limited to, housing stability, mental health, quality of life, and employment outcomes.

#### 2.4.7 What works in inclusion health: an overview of effective interventions for marginalized and excluded populations. (Luchenski et al., 2017)

Luchenski et al. (2017) published a systematic review of reviews that evaluates interventions for marginalized and excluded populations. Although this review covers some of our intended topic areas, such as mental health, case management, housing, and other relevant interventions, it is not exclusive to the homeless population and includes other populations such as those with substance use disorders, prisoners, and sex workers. Our review will be homeless and vulnerably housed population‐specific and will report on a variety of different outcomes.

Our systematic review will serve to inform policy and practice for medical and nursing professional organizations as well as health and social service organizations. The medical complexity and mobility of populations experiencing homelessness and vulnerable housing conditions demand the development of an evidence‐based platform for primary care delivery that will foster trust, partner with community agencies, and train and support front line providers on the complexities of caring for those experiencing homelessness and vulnerable housing (Hwang & Burns, 2014; McNeil, Guirguis‐Younger, & Dilley, 2012; White & Newman, 2015). Furthermore, despite ample published literature on the effectiveness of interventions in populations experiencing homelessness and vulnerable housing, there is a need to synthesize a global evidence base globally. As such, this systematic review will not only contribute towards supporting a global evidence base on homeless research but will also serve to inform the creation of a Canadian guideline document on the health care of populations experiencing homelessness and vulnerable housing. This guideline document will be informed by a separate guideline panel and could serve useful for other countries with health care systems similar to that of Canada.

## 3 OBJECTIVES

The objective of this systematic review is to identify, appraise, and synthesize the best available evidence on the effectiveness and cost‐effectiveness of interventions to improve health, housing stability, and social outcomes for people experiencing homelessness and those who are vulnerably housed. Our outcomes of interest include housing stability, mental health, quality of life, substance use, hospitalization, employment, and income. The following research questions are developed to guide the formation of the systematic review:

1. What are the effects of permanent supportive housing models (i.e., HF) on the health outcomes of people experiencing homelessness and those who are vulnerably housed compared to treatment as usual or no housing?
2. What are the effects of case management and care coordination (i.e., non‐ICM and peer support) on the health outcomes for people experiencing homelessness and those who are vulnerably housed compared to treatment as usual or no care?
3. What are the effects of income assistance (i.e., direct and indirect) on the health outcomes for people experiencing homelessness and those who are vulnerably housed compared to treatment as usual or no support?
4. What are the effects of mental health interventions (i.e., ACT, ICM, and injectable antipsychotics) on the health outcomes for people experiencing homelessness and those who are vulnerably housed compared to treatment as usual or no intervention?
5. What are the effects of addictions interventions (i.e., SCFs, MAPs, and pharmacological interventions for opioid use disorders) on the health outcomes for people experiencing homelessness and those who are vulnerably housed compared to treatment as usual or no intervention?
6. What are the economic considerations while implementing these interventions to improve health, health services, and social outcomes for people experiencing homelessness and those who are vulnerably housed?

## 4 METHODS

### 4.1 Criteria for considering studies for this review

#### 4.1.1 Types of studies

We have followed the Preferred Reporting Items for Systematic Reviews and Meta‐analyses (PRISMA‐P) for systematic review protocols (Moher, Liberati, Tetzlaff, & Altman, 2009).

All study designs must evaluate the effects of an intervention, and have measured outcomes. We will include relevant randomized control trials as they represent the best study design to evaluate the effectiveness of many interventions. We are aware that this type of study may not always be available. For this reason, we will also consider supplementing our findings with other study designs as recommended by the Cochrane Effective Practice and Organization of Care (EPOC) group which meet our PICO criteria. Additional study designs we will consider are nonrandomized control trials, controlled before‐after studies, interrupted time series studies, and repeated measures studies (Effective Practice and Organisation of Care (EPOC)‐Cochrane, 2015).

Nonrandomized trials place participants in different intervention groups in a nonrandom manner. A controlled before‐after study makes observations before and after an intervention is implemented, in both the intervention and control group. These studies have both intervention and control groups but are not be randomized. We will include studies where the data collection is simultaneous in the two groups and the measurement is done using the same methods. Interrupted time series is a study design in which observations are made at multiple time points before and after an intervention. The aim is to determine whether the intervention has a significant effect. We will exclude any studies that do not provide a definite time when the intervention occurred and do not have at least three data points before and three points after the intervention as used by the EPOC Group (Effective Practice and Organisation of Care (EPOC)‐Cochrane, 2015). For repeated measures studies, measurements are calculated from the same group at each time point.

In order to assess the quality of studies, we will use the recommended EPOC critical appraisal criteria (Cochrane EPOC, 2017). Furthermore, all studies reporting relevant outcomes will be assessed using the GRADE approach.

#### 4.1.2 Types of participants

The review will examine interventions targeting people with lived experience of homelessness, defined as those who lack stable, permanent, appropriate housing, or who may be without immediate prospect, means, and ability to acquire it. Such physical living situations can include emergency shelters or provisional accommodations (Canadian Observatory on Homelessness, 2017). Studies must report if participants are homeless or vulnerably housed in order to be included in this review. We will include studies which only have a subset of the sample as homeless or vulnerably housed as long as 50% of the participants were homeless or vulnerably housed. The Delphi consensus process identified several populations in need of additional research considerations (Women, Youth, Indigenous Peoples, immigrants, people with disabilities). We therefore will consider subgroup analysis if our included studies report unique findings on these selected populations. Moreover, our Delphi process identified Indigenous homelessness as a complex priority issue, thus an independent team will lead an Indigenous‐specific project to evaluate the effectiveness of interventions specifically targeting this marginalized population.

#### 4.1.3 Types of interventions

We will include studies that have multicomponent interventions as long as one of the interventions applies to those mentioned above.

All included interventions must be compared to inactive control (i.e., a placebo, no treatment, standard care) or active control intervention (alternative or variant of the intervention; Effective Practice and Organisation of Care (EPOC)‐Cochrane, 2015). In the case in interrupted time series, the control will be a historical control with three data points. If a study is included with more than two intervention arms we will only include the intervention and control arms that meet the eligibility criteria.

#### 4.1.4 Types of outcome measures

Studies must use validated measures and must report at least one of the following outcomes in order to be included in this review. We expect to capture any potential benefits and harms within our outcomes.

Primary outcomes

1. Housing stability: Any measures assessing participants housing status, such as the number of days in stable housing, number of days homeless (on the street or in shelters), number of participants in stable housing, and number of participants homeless (on the street or in shelters). Typical tools to measure housing stability include the Residential Timeline Followback Inventory RTLFB (Tsemberis, McHugo, Williams, Hanrahan, & Stefancic, 2007).
2. Mental health: Any measures assessing psychological status and wellbeing, including but not limited to, psychological distress, self‐reported mental health status, or mental illness symptoms. Typical tools to measure mental health include the Colorado Symptom Index CSI (Boothroyd & Chen, 2008), and the self‐reported mental status SF‐12 (Nelson, Rae, & Townley, 2012).
3. Quality of life: which include an assessment of well‐being, life and personal satisfaction, quality of social relationships, and any specific physical or mental quality of life measures. Typical tools to measure the quality of life include the Lehman Quality of Life Interview (Lehman, 1996), and the EuroQoL 5‐D scale (Lamers, Bouwmans, van Straten, Donker, & Hakkaart, 2006)
4. Hospitalization: Any measures of participants’ use of hospital and emergency services, such as the number of days hospitalized or number of visits to the emergency department. Such measures can be assessed through a standard questionnaire asking participants for their service use, or through accessing the hospital data sets.
5. Substance use: As measured by the number of days using alcohol or substance, the rate, and frequency of using alcohol or substances, number of days of abstinence from alcohol or substances or physical and mental consequences of using alcohol or substances. Typical tools to measure substance use outcomes include the Global Appraisal of Individual Needs‐Short Screener of Substance Use Problems (Dennis et al., 2006).
6. Income: Any measures of money that participants acquire from different resources, including social assistance, disability benefits, donations, and part or full‐time employment. Such measures can be assessed through a standard questionnaire asking participants for their weekly, monthly or annual income from different sources.
7. Employment: Any measures of employment that participants partake during the study period, including but not limited to, number of days of paid employment, number of employed or unemployed participants, hourly wage, and employment tenure. Such measures can be assessed through a standard questionnaire asking participants about their employment rates during the study period.
8. Economic outcome: Any measures of cost, cost‐benefit, cost‐utility, or incremental cost‐effectiveness ratio. Such measures can be obtained from administrative databases and cost reports. An example of how an individualized program cost could be calculated is dividing the sum of all on‐site operation and services costs (maintenance, utilities, insurance, etc.) by the capacity of the project (Larimer et al., 2009)

**Secondary outcomes**

#### 4.1.5 Duration of follow‐up

All follow‐up durations will be included. The duration of follow‐up will vary based on the interventions.

#### 4.1.6 Types of settings

Interventions to be included are those that take place in any setting where the primary care of people with lived experience of homelessness takes place. Primary care is known as the “entry point to the larger health care system” (Tarlier DeniseDefin, 2007) and can be provided by professionals from many disciplines such as family physicians, psychiatrists, social workers, emergency physicians, etc. We also will include community‐based interventions provided in social service or shelter/supervised consumption locations, private or nonprivate clinics, hospital emergency rooms, outreach care, street patrols, mobile care units, etc.

We will exclude all studies conducted in a tertiary care setting and low‐ and middle‐income countries (World Bank, 2019)

### 4.2 Search methods for identification of studies

A search strategy will be developed and peer‐reviewed by a librarian with expertise in systematic review searching. There are no date restrictions or language restrictions set on the search. The search strategy will use a combination of indexed terms, free text words, and MeSH headings such as “homeless or deprived or destitute or impoverished or low income or marginalized or marginalized or needy or poverty or vulnerable”. Refer to Appendix A2 for sample search strategy. The literature search results will be uploaded to Rayyan, a reference manager software package to facilitate the study selection process.

#### 4.2.1 Electronic searches

We will use multiple electronic databases to search for studies, such as Medline, Embase, CINAHL, PsychINFO, Epistemonikos, HTA database, NHSEED, DARE, and Cochrane Central to search for studies on both the effectiveness and cost‐effectiveness of the interventions. We will search for all published studies between the date the database was conceived and the date the search will take place.

#### 4.2.2 Searching other resources

In addition, we will use defined PICO questions and relevant terms to input in a Google search engine to identify the grey literature for published guidelines, experimental and observational articles on evidence relating to our PICO criteria, most notably to identify any missing evidence, particularly for harms. Further, We will consult content experts for any publications or resources that might enrich our findings and screen their suggestions against our inclusion criteria.

### 4.3 Data collection and analysis

#### 4.3.1 Description of methods used in primary research

We will adhere to the review methods as described below:

Phase 1: Conduct a systematic search and selection of studies relating to the effectiveness and cost‐effectiveness of the interventions in each topic section.

**Table:** 

Study characteristics	Inclusion criteria
Population	We will consider peer‐reviewed studies of people with lived experience of homelessness.
Interventions	Interventions for
	Permanent supportive housing (Housing First),
	Care coordination/case management (nonintensive case management and peer support),
	Mental health (assertive community treatment, intensive case management, critical time interventions, and injectable antipsychotics),
	Addictions (supervised consumption facilities, managed alcohol programs, and pharmacological interventions for opioid use disorders),
	Income support (direct and indirect).
Comparison	No intervention, standard intervention, alternative intervention, treatment as usual.
Outcomes	1. Housing stability
	2. Mental health
	3. Quality of life
	4. Hospitalization
	5. Substance use
	6. Income
	7. Employment
	8. Economic outcome
Study characteristics	Primary studies as defined by EPOC criteria (Effective Practice and Organisation of Care (EPOC)‐Cochrane, 2015):
	Randomized controlled trials,
	Nonrandomized controlled trials,
	Controlled before–after studies,
	Interrupted time series and repeated measures studies,
	All study designs must include intervention with a comparison/control group and have measured outcomes.

Study characteristics	Exclusion criteria
	Studies taking place in low‐middle‐income countries (World Bank, 2019),
	Studies that exclusively report on Indigenous‐specific interventions.

We will exclude all studies conducted in low and middle‐income countries an as the heterogeneity in health and social care infrastructure and the differences in family and community support systems significantly impact how homelessness is perceived and managed in such settings (Smartt et al., 2019). We will exclude studies that are exclusive to Indigenous Peoples as the analysis of interventions tailored to this population will be covered by an Indigenous research group. We will not exclude studies based on language or date of publication.

Phase 2: Study screening

Before the beginning of the screening process, two reviewers will undergo a calibration exercise to ensure consistency where they screen a common set of citations and discuss discrepancies. Once reviewers are trained, they will work independently in duplicate to screen the titles and abstracts of all retrieved citations to identify the eligible reviews. Next, the full texts of potentially eligible citations will then be retrieved and screened independently in duplicate. The reviewers will compare the results and resolve disagreements by discussion or with the help of a third reviewer. We will contact authors of reviews once for missing information.

#### 4.3.2 Criteria for determination of independent findings

If an intervention program is evaluated by more than one publication and is published in multiple reports we will only include the most recent report or any additional report with unique outcome assessments (e.g., secondary analysis reports). We will analyze the reports as separate studies so long as they meet all of our PICO criteria. We will analyze studies that report multiple effect sizes for different outcome constructs separately.

#### 4.3.3 Selection of studies

Once studies are screened for PICO criteria they will be hand searched for relevant secondary analysis. Relevant systematic reviews and reference lists will also be hand searched for any additional primary studies which meet our PICO inclusion criteria.

#### 4.3.4 Data extraction and management

We will develop a standardized extraction sheet for each topic variable and create a table of characteristics to describe a summary of findings from our included studies. Please refer to Appendix A3 for sample data extraction sheet. The data extraction sheet will be piloted by two independent reviewers. We will collect and utilize all relevant numerical data (standard deviation, effect estimates, confidence intervals, test statistics, *p* values, etc.). A calibration exercise for data extraction will be performed by reviewers to ensure consistency. Teams of two reviewers will extract data in duplicate and independently. The reviewers will compare results and resolve disagreements by discussion or with help from a third reviewer.

Furthermore, all studies reporting relevant outcomes will be assessed using the GRADE method to appraise the quality and certainty of evidence to determine the validity and effectiveness.

The rating is based on an assessment of

1. Risk of bias (methodological limitation);
2. Inconsistency (heterogeneity) in the direction and/or size of the estimates of effect;
3. Indirectness of the body of evidence to the populations, interventions, comparisons, and/or outcomes;
4. Imprecisions of results (few participant/events/observations and/or wide confidence intervals);
5. Other considerations (effect size and publication bias).

The quality of evidence may be downgraded if there are serious or very serious concerns related to any of the GRADE criteria. All key data will be entered into the GRADEpro software (GRADEpro, 2015). This software will be used to produce GRADE evidence profile tables and summary of findings tables. See Appendix A4 for sample GRADE summary of findings table.

##### Dissemination

Researchers and community scholars will present the findings at research rounds and related conferences for policymakers, practitioners, and lay audience. Newsletters with updates on our project will also be disseminated (i.e., to homeless health networks, researchers, physicians, etc.) Eventually, the findings of this systematic review will contribute to an evidence‐based guideline for primary care practitioners. This document aims to inform primary care practitioners and build a knowledge network around the recommendations and resources for caring for people with lived experience of homelessness. We plan to publish this guideline in as an open‐access document in the Canadian Medical Association Journal and develop an easy to use App to increase dissemination.

#### 4.3.5 Assessment of risk of bias in included studies

EPOC critical appraisal criteria will be used to assess primary studies (Higgins, Sterne, Savović, Page, & Hróbjartsson, 2016). Two reviewers will independently assess the quality of each study in duplicate and the disagreements will be resolved by discussion or using a third reviewer.

#### 4.3.6 Measures of treatment effect

We will analyze studies that report multiple effect sizes for different outcome constructs separately. When we will need to evaluate a single effect size from multiple reported effect sizes in a study, we will use synthetic effect sizes. Synthetic effect sizes will average effect sizes before meta‐analysis.

#### 4.3.7 Unit of analysis issues

Several studies are expected to include outcome data for multiple time points. Comparisons will therefore be carried out separately for periods of 6 months and less (short term), six to 18 months (midterm), and 18 months or more (long‐term). When two different measures of an outcome are used in the same study, these measurements are probably not conceptually congruent. Therefore the different measures will be analyzed separately and will be treated as different outcomes. If different measures (e.g., different questionnaire‐based indexes) are used to measure the same outcome (the same construct), then the procedure will depend on the quality of these indexes. The index with higher quality will be preferred to one with lower or unknown quality. If one index is a standardized, validated and internationally well‐known index, and the other is a local and not validated indexed developed by the evaluators, then the former will be chosen and the latter will be dropped.

Multiple intervention groups within a study with one control group will be pooled if appropriate (if they include different individuals) and compared to that control group. Multiple controls groups will only be pooled if appropriate (if they include different individuals)

Given that cost and resource use estimates are sensitive to features of the local context such as local prices, aspects of service organization and delivery, and given that there are currently no agreed quantitative techniques for synthesizing economic data, a narrative synthesis will be undertaken on costs and resource use data. The analysis will report not only measures of resource use and cost, but also who bears the cost or incurs the resource use, and when the cost/resource use is incurred.

#### 4.3.8 Dealing with missing data

We will contact authors once for any missing data. We will use any supplementary data provided by authors in our analysis, or report findings as extracted otherwise.

#### 4.3.9 Assessment of heterogeneity

We recognize that meta‐analyses include a more precise and explicit estimate of intervention effect than an individual result contributing to any pooled analysis (Glass, 1976), thus we will attempt to conduct meta‐analyses when possible. Where meta‐analyses cannot be conducted due to clinical heterogeneity, we will report outcomes as a narrative synthesis. Clinical heterogeneity will be defined as differences in participant characteristics (e.g., sex, age, baseline disease severity, ethnicity, and comorbidities), types or timing of outcome measurements and intervention characteristics and will be assessed by clinical experts. This will enable us to evaluate the intervention effect, that is, the difference of outcomes of two intervention groups treated differently. A fixed‐effect model assumes that the true effect is identical across all studies contributing to a meta‐analysis (Hedges & Vevea, 1998) This assumption, however, is not plausible when examining the effectiveness of complex interventions (Campbell et al., 2000). Therefore, we will seek the use of a random‐effect model when pooling effects throughout this review. Following this procedure will enable us to determine the direction and size of effects and whether the effect is consistent across included studies. Furthermore, we recognize that our outcomes are complex and will be measured differently across studies. For example, housing stability might be measured using the number of days in stable housing or number of participants in stable housing which reflect clinical heterogeneity. Therefore, we will conduct meta‐analyses only when the outcomes of interest are measured using the same metric. Continuous outcomes will be expressed as standardized mean differences and dichotomous treatment effects will be measured as Odds Ratios, and then transformed into another metric if it is needed to interpret the effect size using that metric. In both cases, 95% confidence intervals and exact *p* values will be used.

#### 4.3.10 Assessment of reporting biases

Reporting biases will be captured within the GRADE framework.

#### 4.3.11 Data synthesis

The effectiveness and cost‐effectiveness of each intervention will be reported based on outcomes in the final systematic review. A meta‐analysis will be conducted where possible, otherwise, a narrative synthesis will be used to report results.

#### 4.3.12 Subgroup analysis and investigation of heterogeneity

Statistical heterogeneity will be assessed using *I* ^2^ statistics. Software for statistical analyses will primarily be RevMan 5.3. The Delphi process identified homeless women as a further marginalized population, therefore we will conduct sex and gender‐based subgroup analysis when feasible, as well as subgroup analysis on priority populations such as youth and peoples with disabilities.

#### 4.3.13 Sensitivity analysis

We will assess and discuss methodological design, publication and other biases from our included studies during the critical appraisal phase. If we detect any sample size or methodological quality concerns whilst conducting a meta‐analysis, we will perform sensitivity analyses to explore the impact of each study on the pooled effect estimate. We will not perform moderator analyses.

#### 4.3.14 Treatment of qualitative research

We do not plan to include qualitative research.

### 4.4 Contributions of authors

- Content: K. P., V. S., G. B., V. B., A. A., T. A, C. K., D. P., G. S., S. M. are or have been involved in various research projects for vulnerable populations. V. S., G. B., T. A, G. S., A. A., S. M. especially, have experience in various types of research projects for people experiencing homelessness or who are vulnerably housed. K. T. is a health economist with The Ottawa Hospital. C. L. was involved in the community scholar program developed by C. K. that aims to engage people with lived experience in the research process.
- Systematic review methods: K. P., P. T., T. A., K. T., A. M. have published systematic reviews. K. P., P. T., K. T., and A. M. have extensive experience authoring systematic reviews for developing evidence‐based guidelines. P. T. has authored systematic reviews of educational, health, legal, and social strategies to reduce inequitable inequalities in health in individuals and populations. T. A. has published reviews on housing.
- Statistical analysis: P. T., K. T. provides statistical consultation.
- Information retrieval: C. M., O. M., V. K., S. M., has experience in retrieving and assessing scientific information.

### 4.5 Declarations of interest

Two authors (V. S. and T. A.) have declared potential conflict in their involvement with the At Home/Chez Soi project.

One author (G. S.), has declared potential conflict in her involvement with a managed alcohol program scoping review (albeit focused on hospital implementation)

### 4.6 Preliminary timeframe

March 30, 2020

(Approximate date for submission of the systematic review.)

### 4.7 Plans for updating this review

We will update our search prior to submitting this review for publication to ensure that our findings are up‐to‐date. As well, the reviews will be updated using the PICO question in 4 years. Kevin Pottie will be responsible for updates.

## SOURCES OF SUPPORT

### Internal sources

Financial Support: Inner City Health Associates

Community scholars and other persons with lived experience provided assistance in priority setting for the review.

### External sources

- No sources of support provided

## APPENDIX A

### A1 Excluded interventions

There is a vast array of interventions available for people experiencing homelessness and those who are vulnerably housed; due to limited published literature specific to this population and the scope of the project, we are not able to include all of the interventions related to this topic. We recognize the importance of many other similar interventions. Here are lists of interventions that will not be included in our review:

Mental health and addictions

- integrated dual disorder treatment, abstinence‐contingent housing, integrated primary care interventions for addiction and mental health, and nicotine replacement therapy

Care coordination and case management

- case management of e.g. HIV, TB, Hepatitis C, Outreach models, Respite care

Income assistance

- Contingency management interventions/incentives for following through with care plans

Other

- dental care, eye care

### A2 Sample search strategy

1 vulnerable populations/ poverty areas/

2 ((deprived or destitute? or impoverished or low income or marginalised or marginalized or needy or poverty or vulnerable) adj2 (adolesc$ or child$ or famil$ or men or people or youth? or women)).tw,kf.

3 homeless persons/ homeless youth/ runaway behavior/

4 (homeless$ or runaway?).tw,kf.

5 (temporar$ adj2 (accommodat$ or home? or hous$)).tw,kf.

6 ((based or housed or residen$ or temporar$) adj2 shelter?).tw,kf.

7 or/1–7

8 exp program evaluation/

9 (effectiveness or initiative? or prevent$ or program$ or reduc$ or strateg$ or treatment).tw.

10 or/8–9

11 systematic review/ meta‐analysis/ randomized controlled trial/ controlled clinical trial/ pragmatic clinical trial/ controlled before‐after studies/ interrupted time series analysis/ controlled before‐after studies/ (randomised or randomized).ab,kf.

12 (before adj2 after adj5 (design$ or study or trial)).tw,kf.

13 ((preintervention? or pre‐intervention? or postintervention? or post‐intervention?) adj5 (study or trial)).tw,kf.

14 ((pre‐test or pretest or (posttest or post‐test)) adj2 (design$ or method$ or study or trial)).tw,kf.

15 *economics/ exp *"Costs and Cost Analysis"/ economics, nursing/ economics, medical/ economics, pharmaceutical/ exp economics, hospital/ economics, dental/ exp "Fees and Charges"/ exp budgets/

16 ((budget$ or economic$ or cost or costs or costly or costing or price or prices or pricing or pharmacoeconomic$ or pharmaco‐economic$ or expenditure or expenditures or expense or expenses or financial or finance or finances or financed) adj6 (analys$ or analyz$ or effect$ or evaluat$ or impact$)).ab. /freq = 2

17 (cost$ adj2 (effective$ or utilit$ or benefit$ or minimi$ or analy$ or outcome or outcomes)).ab,kf.

18 (value adj2 (money or monetary)).tw,kf.

19 exp models, economic/ economic model$.ab,kf.

### A3 Sample data extraction sheet

**Table:**

Bibliographic details	Author	
Year		
Title		
Publication information	Journal name, volume, issue, page numbers, and doi.	
Methods	Population	Total number, setting, diagnostic criteria, age, sex, country, socio‐demographics, comorbidity, and exclusion.
Setting	Description of geographic context (country, city), and intervention context (e.g., primary care setting).	
Study objective	As reported in the study.	
Study methodology	Study design, study duration, and sample size calculation/stat power.	
Sample size and characteristics	How many participants were included, what was the response rate/lost to follow up, and characteristics of the population (e.g., age, sex, education, employment).	
Length of follow‐up	If applicable	
Intervention description	Describe the intervention(s) included in the study. What is implemented, what is the total number of implementation groups, what is the comparison, how is it done, by whom, for whom, etc.	
Findings	Outcomes	Outcomes collected, Outcome definition, and unit of measurement.
End numbers	Number of participants in each group, sample size, and missing participants.	
Results	Summary data for each intervention group.	
Conclusions	Evidence summary	Author's conclusions.
Source of funding	Source of funding and the role of the funder.	
Other information	Ethical considerations, if applicable, other.	

### A4 Sample summary of findings table, GRADE

**Table:**

Certainty assessment							No of patients		Effect		Certainty	Importance
No of studies	Study design	Risk of bias	Inconsistency	Indirectness	Imprecision	Other considerations	Intervention	Comparison	Relative (95% CI)	Absolute (95% CI)		
First outcome (Study Author, Study Year)												
Second outcome (Study Author, Study Year)												
